# Momelotinib: an emerging treatment for myelofibrosis patients with anemia

**DOI:** 10.1186/s13045-021-01157-4

**Published:** 2022-01-19

**Authors:** Helen T. Chifotides, Prithviraj Bose, Srdan Verstovsek

**Affiliations:** Department of Leukemia, Unit 428, The University of Texas MD Anderson Cancer Center, 1400 Holcombe Blvd., Houston, TX 77030 USA

**Keywords:** ACVR1 inhibitor, Anemia, Hepcidin, Iron homeostasis, JAK1/2 inhibitor, Momelotinib, MOMENTUM, Myelofibrosis, Myeloproliferative neoplasm (MPN), Transfusion-independence

## Abstract

The suite of marked anemia benefits that momelotinib has consistently conferred on myelofibrosis (MF) patients stem from its unique inhibitory activity on the BMP6/ACVR1/SMAD and IL-6/JAK/STAT3 pathways, resulting in decreased hepcidin (master iron regulator) expression, higher serum iron and hemoglobin levels, and restored erythropoiesis. Clinical data on momelotinib from the phase 2 and the two phase 3 SIMPLIFY trials consistently demonstrated high rates of sustained transfusion-independence. In a recent phase 2 translational study, 41% of the patients achieved transfusion independence for ≥ 12 weeks. In the phase 3 trials SIMPLIFY-1 and SIMPLIFY-2, 17% more JAK inhibitor-naïve patients and two-fold more JAK inhibitor-treated patients achieved or maintained transfusion independence with momelotinib versus ruxolitinib and best available therapy (89% ruxolitinib), respectively. Anemia is present in approximately a third of MF patients at diagnosis, eventually developing in nearly all patients. The need for red blood cell transfusions is an independent adverse risk factor for both overall survival and leukemic transformation. Presently, FDA-approved medications to address anemia are lacking. Momelotinib is one of the prime candidates to durably address the critical unmet needs of MF patients with moderate/severe anemia. Importantly, momelotinib may have overall survival benefits in frontline and second-line MF patients. MOMENTUM is an international registration-track phase 3 trial further assessing momelotinib’s unique constellation of anemia and other benefits in second-line MF patients; the results of the MOMENTUM trial are keenly awaited and may lead to regulatory approval of momelotinib.

**Graphical abstract:**

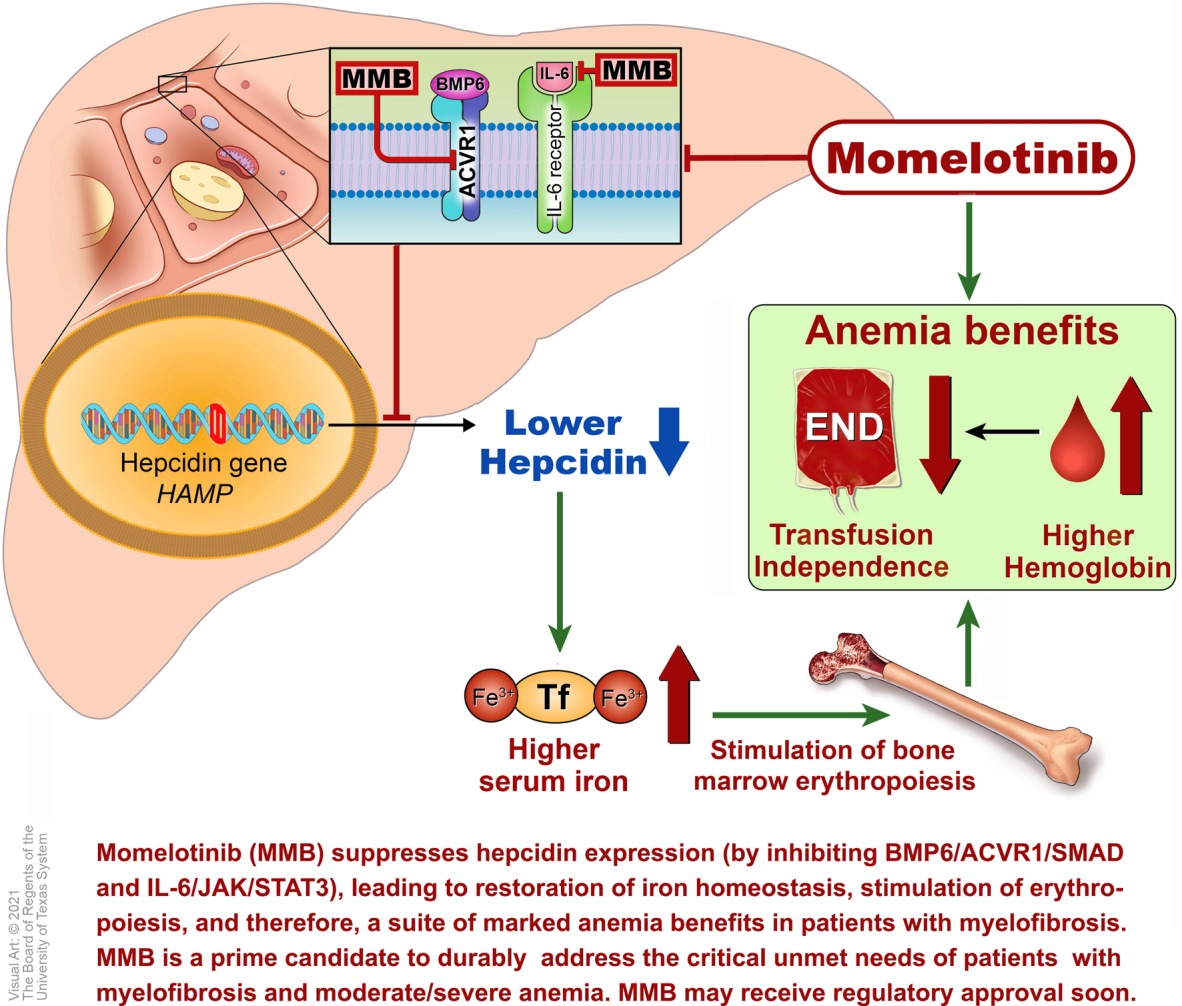

## Introduction

Primary myelofibrosis (PMF) is the most aggressive of the Philadelphia chromosome-negative myeloproliferative neoplasms, a group of closely related, clonal, chronic malignancies of the bone marrow/blood [1] that transform to acute myeloid leukemia (AML) in about 20% of cases [2, 3]. Among other features, myelofibrosis (MF) is characterized by pathologic proliferation of pluripotent stem and progenitor cells, release of pro-inflammatory cytokines by clonal myeloid cells, extramedullary hematopoiesis, splenomegaly, and progressive bone marrow fibrosis [1, 4]. These processes disrupt the physiologic medullary erythropoietic environment, leading to decreased erythropoiesis, progressive bone marrow failure and anemia (hemoglobin [Hb] levels < 10 g/dL); anemia is one of the three cardinal features of MF besides splenomegaly and constitutional symptoms (night sweats, low-grade fevers, itching, bone pain, fatigue, unintentional weight loss, and cachexia) [4]. The seminal discovery of the *JAK2* V617F mutation and the role of the JAK—signal transducer and activator of transcription (JAK—STAT) pathway in MF pathogenesis [5, 6] along with the ensuing clinical development of ruxolitinib has had dramatic results in terms of improving the patients’ quality of life, splenomegaly, systemic symptoms [7, 8], and survival [9, 10]. Ruxolitinib, the first approved JAK1/2 inhibitor for treatment of MF, transformed the therapeutic landscape and has become a global standard of care [7]. However, the incurable (other than by successful allogeneic hematopoietic cell transplantation) nature of the disease (median survival is ≈ 6 years in PMF but it can be much shorter in high-risk patients [11]), potential progression to AML, and the unmet clinical needs of certain cohorts of patients have fueled the quest for novel MF treatments [7, 12–16].

### Anemia in MF

In MF, anemia stems from multiple factors that are mutually related and only partially understood [17]. Besides constitutive activation of the JAK—STAT pathway and dysregulated inflammatory cytokine production leading to inhibition of bone marrow erythropoiesis, sequestration and destruction of circulating erythrocytes by the enlarged spleen is one of the factors contributing to the pathogenesis of anemia in MF patients. At diagnosis of PMF, Hb levels already are below 10 g/dL in about one third of the patients [18–21]; eventually, nearly all patients become anemic [20]. Another study of 1,000 patients with PMF seen at the Mayo Clinic showed that more than 50% of the patients were anemic when referred to the institution, and approximately 25% of them required red blood cell (RBC) transfusions at diagnosis; one year after diagnosis, nearly half of the patients required RBC transfusions [22]. Eventually, nearly all MF patients require RBC transfusions [22], a situation that remains unavoidable during the advanced stages of MF. Notably, RBC transfusion-dependence is one of the risk factors not only associated with inferior survival, but it also portends leukemic transformation [2, 3].

Anemia and RBC transfusion dependence constitute key adverse prognostic factors in MF that are inversely associated with quality of life [16, 17] and survival [21–24]. The risk of death was 1.5-fold higher in severely anemic, transfusion-dependent MF patients compared to that in moderately anemic patients [24]. The necessity for RBC transfusions post-splenectomy was also associated with inferior survival in MF patients [25]. Anemia is a prime correlate of progressive disease in MF patients; consequently, MF-related anemia, especially transfusion-requiring anemia, is one of the most important disease consequences to address. Furthermore, transfusion-requiring anemia is a tremendous burden for patients and healthcare systems. Anemia (of varying severity) is an independent adverse risk factor in the International Prognostic Scoring System (IPSS), the Dynamic International Prognostic Scoring System (DIPSS), the Mutation-Enhanced International Prognostic Scoring System 70 (MIPSS70) and MIPSS70-plus for PMF, and the Myelofibrosis SECondary to PV and ET-Prognostic Model (MYSEC-PM) for secondary MF [11]. In the DIPSS, which can be applied at any time during the course of the disease, Hb < 10 mg/dL carries two-fold higher weight (2 points instead of 1) compared to the other four risk factors (age, white blood cell count, constitutional symptoms, circulating blasts) [21]. In the DIPSS-plus model, the need for transfusions is considered an additional prognostically adverse risk factor that increases the score (over that based on the DIPSS category) by another point, thereby automatically pushing severely anemic patients (i.e., transfusion-requiring) into the intermediate-2 category (with a median survival of about 3 years) regardless of other risk factors [26]. In a recent study, stratification of 1,109 MF patients by grade of anemia demonstrated that patients with severe (Hb < 8 g/dL or transfusion dependence) and moderate (Hb in the range 8–10 g/dL) anemia had a median survival of 2.1 and 3.4 years, respectively [24]. Univariate analysis of the data from the same study demonstrated that the hazard ratio (HR) for severe and moderate anemia was 3.4 and 2.1, respectively [24]. Notably, the cumulative incidence of anemia was lower in PMF patients harboring the driver *CALR* mutation versus *JAK2*- and *MPL*- mutants; conversely, “triple negative” patients for the three driver mutations manifested the highest cumulative incidence of anemia [27]. In another study of 722 patients with PMF, disease-related anemia also showed a significant association with driver mutation status and the non-driver mutation *U2AF1* (30% and 18% of the patients harboring mutated *U2AF1* had severe and moderate anemia, respectively) on univariate analysis [28].

### Anemia management and JAK inhibitors

In MF patients, disease-related anemia can be exacerbated by treatment with ruxolitinib because of myelosuppression, an adverse event that is consistent with the drug’s interference with erythropoietin signaling via JAK-STAT (especially JAK2), which is essential for erythropoiesis [5]. An exploratory analysis of the pooled 3-year data for patients who were enrolled in the ruxolitinib arms of the two randomized phase 3 clinical trials COMFORT-I [29] and COMFORT-II [30] showed dose-dependent anemia: mean Hb levels reached a nadir in the first 8–12 weeks and subsequently recovered to a new, lower baseline by week 24 [31, 32]; and RBC transfusions increased during the first 8–12 weeks of treatment [33, 34]. Importantly, an analysis by Gupta and colleagues demonstrated that post-baseline ruxolitinib-induced anemia did not decrease overall survival (OS), both in subgroups with and without baseline anemia, as opposed to the deleterious effects of disease-related anemia in MF patients [33, 34]. On the contrary, treatment with ruxolitinib overcame the adverse effects of MF-associated anemia on survival [35]. However, among the patients from the two COMFORT studies who qualified for the exploratory analysis, anemia worsened in 69% of the patients with baseline anemia (< 10 mg/dL) after treatment with ruxolitinib, and 61% of the patients who did not have baseline anemia experienced on-treatment anemia [33, 34]. The investigators of the COMFORT-I and COMFORT-II trials reported new or worsening grade 3/4 anemia in about 46% of the patients treated with ruxolitinib [31, 32].

A more conservative ruxolitinib dosing regimen (10 mg twice daily for the first 12 weeks, followed by dose escalation as tolerated) was administered to MF patients with anemia (Hb < 10 g/dL) in a phase 2 single-arm study (REALISE trial) [36]. Although the conservative regimen had efficacy that was comparable to previous clinical trials with ruxolitinib in MF patients, it is well known from several studies that the ruxolitinib dose is correlated with spleen response and survival, thus favoring dose intensity at the beginning of treatment [7]. As MF evolves, bone marrow failure progresses, leading to impaired erythropoiesis/worsening anemia, and splenomegaly; and constitutional symptoms often become worse [37]. In this setting, increasing the dose of ruxolitinib to treat splenomegaly and symptoms may not be a viable option because it would further accentuate anemia given the essential role of JAK2-mediated erythropoietin signaling in erythropoiesis. In clinical practice, anemia appears to be the leading cause of ruxolitinib discontinuation in MF patients [7, 14, 38, 39], and the rates of discontinuation due to anemia vary widely in clinical practice [38–41]. Besides the primary challenge of anemia, thrombocytopenia and loss of spleen response pose secondary challenges [37], often resulting in ruxolitinib discontinuation [14, 16, 19, 20]. Notably, the median OS of MF patients was 11–14 months after discontinuation of ruxolitinib in several studies [39, 42–45]; in one study, the median OS was 27.5 months when restricting the analysis to patients who discontinued ruxolitinib while in the chronic phase [43].

Fedratinib is the second JAK2 inhibitor that was approved in the US in August 2019 for treatment of intermediate-2 and high-risk MF, providing a viable option for patients with resistance or intolerance to ruxolitinib [7]. Fedratinib induces similar myelosuppression to ruxolitinib because it also interferes with erythropoietin signaling via JAK-STAT. In the phase 3 JAKARTA trial, in which JAK-inhibitor-naïve MF patients were treated with fedratinib, anemia was the most common hematological toxicity; 34% and 75% of the patients developed new or worsening grade 3 anemia at a median of 2 and 3 months after treatment initiation, respectively, and 17% of the patients became transfusion-dependent during treatment [46–48]. In the phase 2 JAKARTA-2 trial wherein MF patients who had been previously treated with ruxolitinib were enrolled, 53% of the patients had Hb < 10 g/dL and 14% were transfusion-dependent at baseline [49]. In the JAKARTA-2 trial, grade 3/4 anemia was the most common hematological adverse event (encountered in 38% of the intention-to-treat population) [49]; 44% of the 79 patients who met “stringent” criteria for ruxolitinib failure in a later re-analysis had grade 3/4 anemia [50].

Pacritinib is a relatively non-myelosuppressive JAK2/interleukin-1 receptor associated kinase 1 (IRAK1) inhibitor in advanced clinical development for patients with severe thrombocytopenia [7]; the approved JAK inhibitors are not recommended for this subgroup of patients because they exacerbate cytopenias. Pacritinib was evaluated in two phase 3 trials, PERSIST-1 [51] and PERSIST-2 [52], which enrolled JAK inhibitor-naïve patients regardless of platelet count and previously treated patients (including those who had previously received ruxolitinib) with platelets ≤ 100 × 10^9^/L at baseline, respectively. In PERSIST-1, the median Hb level increased from 9.1 at baseline to 10.4 g/dL at week 24 in patients treated with pacritinib versus best available therapy (BAT) excluding ruxolitinib; and 9 out of the 36 transfusion-dependent patients (25%) in the pacritinib arm achieved transfusion independence [51]. In PERSIST-2, the RBC transfusion burden was reduced in 19% and 22% of the patients treated with pacritinib once and twice daily at week 24, respectively, versus 9% of the patients treated with BAT (45% was ruxolitinib) [52]. Pacritinib appears to elicit significant spleen responses in patients with the “myelodepletive phenotype” of MF or cytopenic MF, which has been associated with anemia, thrombocytopenia, *JAK2* V617F variant allele frequencies below 50%, and smaller spleen size [53, 54]; efficacy in this subgroup, associated with a poor prognosis, may be attributed to the inhibitory activity of pacritinib on IRAK1 [37].

Allogeneic hematopoietic stem cell transplantation (allo-HSCT) may be considered a potentially curative option for intermediate-2 and high-risk MF patients or for intermediate-1 risk patients with transfusion-dependent anemia [55], but the procedure is limited to a small subgroup of fit patients and has high mortality rates in high- and very high-risk patients according to the MF transplant scoring system [56]. Notably, two studies showed that post-transplant OS was superior in MF patients who underwent allo-HSCT while still responding to JAK inhibitors [57, 58].

Anemia in MF patients is challenging to manage. Immunomodulatory agents (IMiDs®), corticosteroids, erythropoiesis-stimulating agents (ESAs), and androgens (including danazol), both alone and in combination with ruxolitinib have been commonly used to manage anemia [16, 17, 20, 59]. Thalidomide is an immunomodulatory agent that may attenuate cytopenias in MF patients and is non-myelosuppressive at a low dose (50 mg daily) [60–62]. In collaboration with the Memorial Sloan Kettering Cancer Center, we are evaluating thalidomide in combination with ruxolitinib from the outset or in patients who had a suboptimal response to ruxolitinib monotherapy after ≥ 3 months in an investigator-initiated phase 2 trial (NCT03069326) [63]. Danazol is a synthetic steroid that induces anemia responses in about 30% of MF patients with a median response duration of 5 months, but has a lower response rate in transfusion-dependent patients [19]. Danazol is often administered empirically in MF patients with anemia, on ruxolitinib; however, a pilot phase 2 trial assessing danazol in combination with ruxolitinib in MF patients was terminated due to lack of anemia response [64]. In addition, during treatment with danazol, patients should be monitored for potential risks (e.g., transaminitis, prostate cancer). ESAs induce anemia response rates of 40–45%, and favorable responses have been associated with milder anemia (absence of RBC transfusion dependence), and lower erythropoietin levels at baseline [19]. Overall, the efficacy of the aforementioned approaches is limited, and the majority of responses are short-lived [17, 65]. As noted in the preceding section, the requirement for transfusions is an independent adverse factor in the DIPSS-plus model [26]. The necessity for RBC transfusions during the late stages of MF in most patients shows the low effectiveness of the existing treatments for anemia.

The above overview clearly shows that anemia − an important adverse prognostic factor in MF − remains a major challenge and significant unmet clinical need in the management of MF, especially when the patients are transfusion-dependent. The importance of addressing the unmet needs of moderately and severely anemic patients with MF spurred the renewed clinical development of momelotinib, despite previous setbacks, because it elicited noteworthy responses regarding anemia and RBC transfusion-dependence in MF patients while demonstrating comparable efficacy to ruxolitinib in treating the other two cardinal features of MF (splenomegaly and constitutional symptoms). Consequently, momelotinib is uniquely poised to fill the critical gap of addressing anemia in MF patients.

### Anemia benefit of momelotinib achieved by inhibiting ACVR1, suppressing hepcidin expression, and mobilizing iron

Momelotinib is a small-molecule oral inhibitor of the JAK1/JAK2 kinases (JAK1, IC_50_ = 26.9 nM and JAK2, IC_50_ = 1.4 nM) with potent inhibitory activity against the type 1 kinase activin A receptor or activin receptor-like kinase-2 (ACVR1/ALK2, IC_50_ = 8.4 nM) [66]. Momelotinib’s potent and unique (among currently approved and emerging JAK inhibitors) inhibition of hyperactivated ACVR1/ALK2 signaling underlies the suppression of aberrant activation of hepcidin transcription in the liver, increase in circulating iron and hemoglobin, stimulation of erythropoiesis, and its consequent marked benefit on iron-restricted anemia, including reversal of RBC transfusion-dependence in MF patients [67].

ACVR1/ALK2 is a bone morphogenetic protein (BMP) type-1 receptor and a key regulator of hepcidin production on the surface of hepatocytes. BMPs belong to the transforming growth factor-beta (TGF-β) superfamily of cytokines. The BMP signaling pathway has important functions in adult tissue homeostasis and is known to be the driver of inflammatory diseases in different organ systems, including anemia of inflammation or chronic disease [68, 69], which is mediated by elevated hepcidin levels. 

**Fig. 1:**
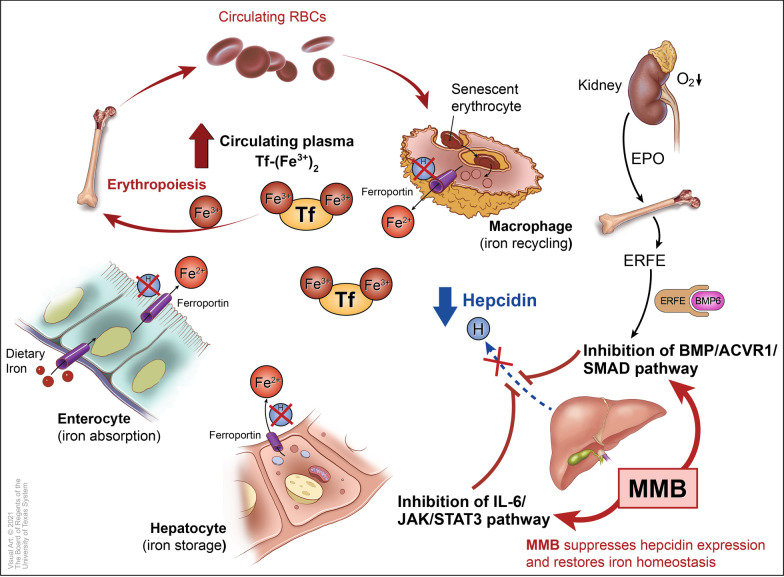
Systemic iron homeostasis and its regulation by the hepcidin-ferroportin axis. Momelotinib suppresses hepcidin expression in the liver— via inhibition of the BMP6/ACVR1/SMAD and IL-6/JAK/STAT3 pathways— leading to an increase in circulating iron and hemoglobin levels; and restoration of erythropoiesis. Abbreviations: *ACVR1* activin A receptor type 1, *BMP* bone morphogenetic protein, *EPO* erythropoietin, *ERFE* erythroferrone, *H* hepcidin, *IL-6* interleukin-6, *JAK* Janus kinase, *MMB* momelotinib, *RBCs* red blood cells, *SMAD* small- mothers-against-decapentaplegic, *STAT3* signal transducer and activator of transcription 3, *Tf* transferrin

Hepcidin, a hepatic-secreted small peptide (25 amino acids) hormone, is the master regulator of iron homeostasis [70]. Hepcidin is a negative regulator of systemic iron levels (serum iron and hepcidin levels are inversely related); and its regulation is controlled by a feedback mechanism involving plasma iron levels, thus demonstrating the key role of hepcidin in both iron absorption and release of recycled iron from macro-phages (Fig. 1) [70].

**Fig. 2:**
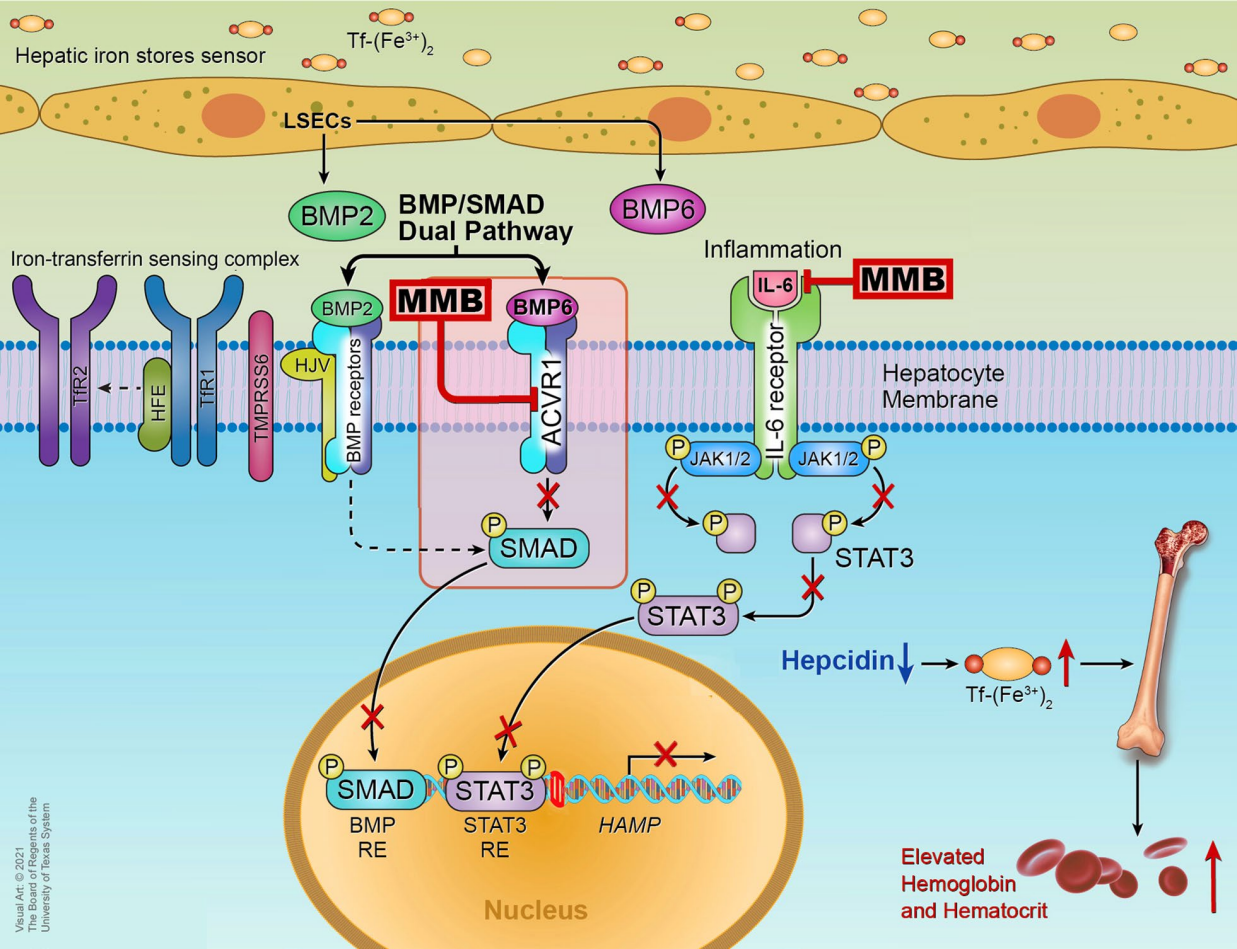
Momelotinib suppresses hepcidin expression in hepatocytes via inhibition of the BMP6/ACVR1/SMAD and IL-6/JAK/STAT3 pathways. Hepcidin is elevated in MF patients due to aberrant hyperactivation of the BMP6-stimulated kinase ACVR1/ALK2 signaling and inflammatory cytokine signaling via IL-6, which is also elevated in MF patients. Suppression of hepcidin expression in the liver increases circulating iron [Tf-(Fe^3+^)_2_] and hemoglobin; and stimulates erythropoiesis in the bone marrow. Abbreviations: *ACVR1* activin A receptor type 1,  *BMP* bone morphogenetic protein, *BMP2* bone morphogenetic protein 2, *BMP6* bone morphogenetic protein 6, *BMP RE* BMP response element, *HAMP* hepcidin gene, *HFE* homeostatic iron regulator, *HJV* hemojuvelin, *IL-6* interleukin-6, *JAK* Janus kinase, *LSECs* liver sinusoidal endothelial cells, *MMB* momelotinib, *SMAD* small-mothers-against-decapentaplegic, *STAT3* signal transducer and activator of transcription 3, *STAT3 RE* STAT3 response element, *Tf* transferrin, *TfR1* transferrin receptor-1, *TfR2* transferrin receptor-2,* TMPRSS6* Transmembrane protease, serine 6

Hepcidin production is upregulated by two major molecular pathways that are strictly interconnected:

a) cytokine-driven ACVR1 signaling. BMP6 is the predominant endogenous regulator of hepcidin expression and iron metabolism [71]. The BMP/small-mothers-against-decapentaplegic (SMAD) proteins pathway has a central role in hepcidin synthesis [72, 73]. The dual-branch BMP pathway is initiated by BMP6 and BMP2 binding to the BMP receptors (ACVR1/ALK2 and ALK3, respectively) on the surface of hepatocytes, triggering phosphorylation, downstream activation of SMAD1/5/8, translocation to the nucleus (after interaction with SMAD4), and initiation of hepcidin transcription (activation of the BMPs/BMP Receptors/SMAD1/5/8 signaling pathway) (Fig. 2) [73, 74]. BMP6 expression is iron-dependent (BMP6 synthesis increases when iron levels are elevated), whereas BMP2 is less sensitive to iron levels; both BMP6 and BMP2 are produced in the liver sinusoidal endothelial cells (LSECs), which are non-parenchymal, highly permeable hepatic cells lining the sinusoidal capillary channels of the liver (LSECs are surrounded by hepatocytes). The receptor ACVR1/ALK2 plays an essential role in this process (BMP6/ACVR1/ SMAD1/5/8 iron-sensing pathway). The BMP co-receptor hemojuvelin (HJV), the homeostatic iron regulator (HFE)  protein, and the diferric transferrin sensor transferrin receptor 2 (TfR2) are necessary for hepcidin regulation in response to changes in iron levels [73, 75–78].

b) inflammatory cytokine signaling primarily via interleukin-6 (IL-6), a key hepcidin-inducing cytokine produced during inflammation [79, 80]; IL-6 acts through the JAK-STAT3 pathway in hepatocytes (Fig. 2) [73, 76, 77]. During inflammation, IL-6 is released and binds to its receptor on the hepatocellular membrane, inducing JAK1/2 to phosphorylate STAT3 and activate hepcidin transcription. However, it has been shown that complete hepcidin induction via the IL-6/JAK/STAT3 pathway is associated with integrity of the BMP/ SMAD1/5/8 pathway (a threshold of BMP6/SMAD signaling is required); and IL-6 − mediated expression of hepcidin requires binding of the dimerized phosphorylated STAT3 (pSTAT3) to the STAT binding site in the hepcidin promoter [73, 74, 76, 77, 81]. While IL-6−induced synthesis of hepcidin, as a response to infection, is considered a general host defense mechanism against iron-dependent microorganisms, persistent inflammation and high hepcidin levels result in sequestration of iron in the macrophages of the reticuloendothelial system and reduced duodenal iron absorption, dysregulated iron homeostasis, decreased iron available for erythropoiesis and thus, anemia of chronic disease [72].

Hepcidin’s inhibitory effect on iron export from the cells is mediated by interacting directly with ferroportin. Ferroportin is a transmembrane iron efflux transporter that is found on iron-releasing cells (splenic macrophages that recycle iron of senescent erythrocytes, duodenal enterocytes that absorb iron, and iron-storing hepatocytes; Fig. 1). Binding of hepcidin to ferroportin occludes iron efflux from iron-releasing cells and induces internalization and lysosomal degradation of ferroportin, leading to iron sequestration in the cells and hypoferremia (decreased concentrations of circulating iron), which is characteristic of anemia of chronic disease. Conversely, suppression of hepcidin under erythropoietic stimuli (for example, erythropoietin-stimulated erythroblasts in the bone marrow release erythroferrone, which suppresses hepcidin expression by sequestering BMP6 [82], thus promoting erythropoiesis; Fig. 1) or hypoxia leads to release of sequestered iron from cellular stores into the plasma via the cellular exporter ferroportin [76, 77], binding of transferrin (Tf) to iron [Tf-(Fe^3+^)_2_], and increased concentrations of iron in the circulation. Transferrin, the main iron carrier protein in the plasma, transports iron between sites of recycling, absorption and storage in the bone marrow erythroblasts for heme and hemoglobin synthesis, and RBC production (Fig. 1). Therefore, the hepcidin-ferroportin axis has a tight influence on erythropoiesis, and thereby, the pathophysiology of anemia in the setting of hepcidin dysregulation, for example, iron-restricted erythropoiesis due to aberrantly elevated hepcidin [74]. Consequently, agents that inhibit hepcidin expression [77]—such as momelotinib, for example − can restore iron homeostasis, induce the release of stored iron from the macrophages of the reticuloendothelial system and make it available for RBC production in the bone marrow (Fig. 1).

In accordance with momelotinib-driven inhibition of ACVR1/ALK2, induction of phosphorylated SMAD1/5/8 decreased in BMP6-stimulated HepG2 cells when momelotinib was added [66]. In addition, reduced amounts of phosphorylated SMAD1/5/8 (pSMAD1/5/8) were found in hepatic nuclear extracts; and a dose-dependent decrease in hepcidin expression, higher serum iron availability, and increased erythropoiesis were noted in a group A *Streptococcus* peptidoglycan-polysaccharide fragment–induced rat model of anemia of chronic disease treated with momelotinib for 3 days [66]. Furthermore, consistent with the BMP receptor ACVR1-mediated inhibition of hepcidin expression, treatment of transfusion-dependent MF patients with momelotinib in a translational phase 2 study induced an acute decrease of hepcidin levels 6 h after the first dose and at every study visit. Hepcidin levels declined over the 24 weeks of momelotinib administration; in 14 patients (34%) who became transfusion-independent by week 24, the median hepcidin level decreased from 23 nM (pre-transfusion independence level) to ~ 9 nM at week 24 [67]. The decrease in serum hepcidin levels led to restoration of iron homeostasis (serum iron levels increased by 61 µg/dL at 2 weeks compared to baseline in these 14 patients). In these patients, erythropoiesis was stimulated, and a sharp increase of Hb, which continued there-after, was noted by week 2 [67].

Notably, in another study, considerably higher hepcidin levels were detected in the plasma of patients with PMF (median 156,28 pg/mL) compared to normal controls (median 13,45 pg/mL), and they were associated with anemia, RBC transfusion-dependence and significantly inferior survival [83]. Zhou and colleagues reported elevated levels of hepcidin and IL-6 in patients with primary and secondary MF [84]. In this study, hepcidin levels remained markedly elevated in a subgroup of MF patients treated with ruxolitinib [84]; these findings corroborate the differentiated activity of momelotinib via inhibition of BMP6/ACVR1/ SMAD1/5/8-mediated hepcidin expression as compared to ruxolitinib, which does not inhibit this pathway [ACVR1, IC_50_ (rux) = 6100 nM] [66]. As reported by Verstovsek and colleagues, however, IL-6 levels decreased compared to baseline in patients with primary and secondary MF who were treated with ruxolitinib in the phase 1/2 trial [85]. In line with the higher hepcidin levels and aberrant hyperactivation of the BMP6/ACVR1/SMAD signaling pathway in MF patients, higher levels of BMP6 expression were recorded in patients with advanced MF; and BMP6 and BMP receptor-2 were considerably overexpressed in the bone marrow of patients with prefibrotic PMF compared to controls [86]. Furthermore, in another study, Garimella and colleagues reported significant amounts of BMP2, BMP6 and their receptors, released by megakaryocytes in the bone marrow of GATA-1^low^ mice with myeloproliferative neoplasms; the authors suggested that BMPs and their receptors may play a role in osteoblastic activity and osteosclerosis [87]. In another study, more than 30% of endothelial cells in the small vessels of the bone marrow and spleen from patients with PMF demonstrated a mesenchymal phenotype; in vitro, this process was activated by inflammatory cytokines and was sustained by upregulation of BMP6 [88].

Regarding the effect of momelotinib on elevated inflammatory cytokines, IL-6 levels decreased sharply 6 h after the first dose of momelotinib and remained considerably lower compared to baseline in a phase 1/2 study assessing the agent in MF patients [89]. In another phase 2 clinical trial evaluating momelotinib in transfusion-dependent MF patients, pSTAT3 and hepcidin decreased compared to baseline [67]. Furthermore, similar findings were noted in a preclinical study: namely, IL-6 − induced pSTAT3 was reduced in HepG2 cells and a rat model of anemia of chronic disease after treatment with momelotinib [66]. Conversely, in the same study, ruxolitinib inhibited the IL-6/JAK/STAT3 pathway only but not BMP6/ACVR1/SMAD1/5/8 in the rat model [66]. These findings are consistent with momelotinib’s inhibitory effects on both the BMP6/ACVR1/SMAD1/5/8 and IL-6/JAK/STAT3 pathways [66], with IL-6/JAK/STAT3 playing a secondary role by boosting the BMPs/BMP Receptors/SMAD axis, which drives the required basal signaling for hepcidin expression [73, 74, 76, 77, 81]. The aforementioned findings underscore the unique inhibitory activity of momelotinib on hepcidin expression and amelioration of iron-restricted anemia, thereby promoting erythropoiesis [66] under inflammation (such as in MF patients) as compared to agents that solely inhibit ACVR1 or JAK1/2.

### Momelotinib confers marked anemia benefits on MF patients in phase 1/2 and 3 clinical trials

Momelotinib has been in clinical development for more than 10 years in phase 1, 2 and 3 clinical trials (Table 1). More than 820 patients with MF have been treated with momelotinib while enrolled in clinical trials, including the two phase 3 SIMPLIFY studies [90]; and more than 100 patients who were enrolled in the two SIMPLIFY trials or earlier phase 2 studies continue to be treated with momelotinib in the extended access protocol for 10 years and beyond (as of September 2020) [90, 91].

**Table 1:** Synopsis of clinical trials evaluating momelotinib and the anemia benefits it elicited in MF patients

Study title	ClinicalTrials. gov Identifier	Phase	Population studied	Anemia benefits	Duration	References
A phase 1/2, open-label, dose-escalation study evaluating the safety, tolerability, pharmacokinetics and pharmacodynamics of orally-administered CYT387^a^ in PMF, post-PV MF or post-ET MF	NCT00935987 Core study	1/2	166 intermediate- and high-risk MF patients	Of 41 anemia-evaluable patients: 70% (23/30) became transfusion-independent by IWG-MRT criteria (2006) Median duration of transfusion-independence: 9.6 months	11/2009–04/2012	[92]
An open-label phase 2 extension study evaluating the long term safety, tolerability, and efficacy of orally-administered CYT387^a^ in PMF, post-PV MF or post-ET MF	NCT01236638 Extension of the core study NCT00935987	Extension of core phase 1/2 study	120 intermediate- and high-risk MF patients	Of 111 anemia-evaluable patients: 75% (54/72) and 68% became transfusion-independent (for ≥ 8 weeks) at 8 and 12 weeks, respectively; 28% (11/39) of the patients with Hb < 10 g/dL had a median increase of 2.4 g/dL at 8 and 12 weeks Median duration of 8-week anemia response: 7.7 months	11/2010–06/2014	[93]
Seven-year follow-up study of 100 patients with PMF, post-PV MF or post-ET MF, treated with CYT387^a^ in a phase 1/2 clinical trial	NCT00935987 (part of the larger phase 1/2 trial with the same study identifier)	7-year follow-up of the phase 1/2 trial	100 intermediate- and high-risk MF patients treated at the Mayo Clinic	51% of the patients became transfusion-independent, and 44% of the patients had improvement in anemia	7 years	[94]
A phase 1/2, open-label study evaluating twice-daily administration of CYT387^a^ in PMF, post-PV MF or post-ET MF	NCT01423058	1/2	61 intermediate- and high-risk MF patients	Of 29 transfusion-dependent patients at baseline, 51.7% (15) achieved transfusion-independence for ≥ 8 weeks; 27% (3/11) transfusion independent patients had a Hb increase of ≥ 2 g/dL for ≥ 8 weeks	08/2011–06/2014	[89]
A phase 2, open-label, translational biology study of momelotinib in transfusion-dependent subjects with PMF, post-PV MF or post-ET MF	NCT02515630	2	41 transfusion-dependent MF patients	41% (17/41) of the patients became transfusion-independent^b^ 78% (21/27) of the transfusion-dependent patients had ≥ 50% decrease in RBC transfusion requirements for ≥ 8 weeks 49% transfusion-independence response rate at any time during the study in the evaluable population after ≥ 12 weeks of follow-up	01/2016–08/2017 Extension study: 04/2014–12/2018	[67]
SIMPLIFY-1: A phase 3, randomized, double-blind active-controlled study evaluating momelotinib versus ruxolitinib in subjects with PMF, post-PV MF or post-ET MF	NCT01969838	3 Randomization period: 24 weeks	432 JAK inhibitor-naïve patients who had high-risk, intermediate-2 risk or symptomatic intermediate-1 risk MF and platelet counts ≥ 50 × 10^9^/L	At week 24: 66.5% of the patients in the momelotinib arm became transfusion-independent^c^ versus 49.3% in the ruxolitinib arm (at baseline, 24.7% and 24% were transfusion-dependent, respectively) 83% of the patients treated with momelotinib required ≤ 4 units of RBCs versus 62% on ruxolitinib Median RBC transfusion rate was 0 units/month with momelotinib versus 0.4 units/month with ruxolitinib At week 48: 75% of the patients treated with momelotinib only became transfusion-independent versus 67% of the patients who were in the ruxolitinib arm and crossed over to momelotinib Odds of remaining transfusion-independent were 9.3 times higher for momelotinib-treated patients versus ruxolitinib-treated patients Median duration of transfusion-independence was not reached with momelotinib after follow-up of ≥ 3 years	12/2013–05/2019 Extension study: 05/2018 (onset) Ongoing^f^	[91, 95, 98]
SIMPLIFY-2: A phase 3, randomized study to evaluate the efficacy of momelotinib versus BAT in anemic or thrombocytopenic subjects with PMF, post-PV MF or post-ET MF who were treated with ruxolitinib	NCT02101268	3 Randomization period: 24 weeks	156 anemic or thrombocytopenic patients with MF, post-PV MF or post-ET MF previously treated with ruxolitinib^d^ 104 patients received momelotinib, and 52 patients received BAT (89% ruxolitinib); 56% (58/104) and 52% (27/52) were transfusion-dependent in the momelotinib and BAT/ruxolitinib arm, respectively; no platelet count was set	At week 24: 43% (45/104) of the patients treated with momelotinib became transfusion-independent^c^ versus 21% (11/52) in the BAT/ruxolitinib arm Median RBC transfusion rate was 0.5 units/month with momelotinib versus 1.2 units/month with BAT/ruxolitinib At week 48: 55% of the patients treated with momelotinib from the onset became transfusion-independent versus 40% who were in the BAT/ruxolitinib arm and crossed over to momelotinib During the entire treatment period: 40% (42/104) of the momelotinib-treated patients versus 27% (14/52) in the BAT/ruxolitinib cohort did not require RBC transfusions Median duration of transfusion independence with momelotinib was > 1 year at any time during the study	06/2014–04/2019 Extension study: 05/2018 (onset) Ongoing^f^	[91, 96]
MOMENTUM: A randomized, double-blind phase 3 study of momelotinib versus danazol in symptomatic, anemic subjects with PMF, post-PV MF or post-ET MF, previously treated with JAK inhibitors^e^	NCT04173494	3 Randomization period: 24 weeks	180 patients with MF, post-PV MF or post-ET MF Eligible patients were anemic (Hb < 10 g/dL) and symptomatic (TSS ≥10) at baseline (platelet count ≥ 25 x 10^9^/L); and patients had been previously exposed to an approved JAK inhibitor	Proportion of transfusion-independent^c^ patients at week 24; and cumulative transfusion burden and Hb improvement RBC transfusions will be recorded at 4-week intervals until week 48 and up to the end of week 204 (open label treatment period)	02/2020 Ongoing	[90]

*BAT* best available therapy, *ET* essential thrombocythemia, *Hb* hemoglobin, *IWG-MRT* International Working Group-Myeloproliferative Neoplasms Research and Treatment, *JAK* Janus kinase, *MF* myelofibrosis, *PMF* primary myelofibrosis*, PV* polycythemia vera, *RBC* red blood cell, *TSS* Total Symptom Score

^a^CYT387: former name for momelotinib

^b^Absence of RBC transfusion for ≥ 12 weeks at any time on the study

^c^Absence of RBC transfusion and no Hb level < 8 g/dL in the  12 weeks preceding the end of the randomized treatment period (week 24)

^d^Patients were previously exposed to ruxolitinib for ≥ 28 days and required RBC transfusions while treated with ruxolitinib or a dose reduction to < 20 mg twice daily due to grade 3 or worse anemia, thrombocytopenia or bleeding

^e^Ongoing international, registration-track, pivotal phase 3 trial, sponsored by Sierra Oncology, Inc. (Vancouver, Canada)

^f^Subjects remaining on momelotinib treatment from studies NCT01969838, NCT02101268, NCT02124746, and NCT04173494 were permitted to enroll in an additional extended access study (NCT0344113), which is currently ongoing

In line with its ACVR1-mediated inhibition of hepcidin expression, resulting in iron mobilization from cellular stores and enhanced erythropoiesis (Fig. 1), momelotinib has provided a constellation of important benefits with respect to anemia in MF patients. Anemia benefits included conversion of transfusion-dependence at baseline to sustained transfusion-independence, substantive reductions in transfusion burden, and high rates of transfusion-independence, frequent elevation of Hb ≥ 1 g/dL; and fewer adverse events of anemia in the momelotinib arms of late phase clinical trials.

The first phase 1/2 clinical trial assessing momelotinib in intermediate-2 and high-risk patients (including intermediate-1 risk) comprised two parts, the dose escalation study (60 patients from the Mayo Clinic) and the confirmation phase of the trial (106 patients from multiple centers) [92]. The core study was followed by an extension phase (120 patients) [93]. Among the patients who could be evaluated for anemia benefits (n = 111) from both phases (core/extension), 75% and 68% of the patients achieved transfusion-independence for 8 and 12 weeks, respectively; and 28% of the patients with Hb < 10 g/dL had an anemia response (median Hb increase 2.4 g/dL) for both 8 and 12 weeks [93]. In the 7-year follow-up study of 100 MF patients treated with momelotinib at the Mayo Clinic, 51% of the patients achieved transfusion-independence, and 44% had improvement in anemia [94]. In another phase 1/2 study assessing twice-daily administration of momelotinib in 61 MF patients (intermediate- or high-risk), 45% of the patients showed an overall anemia response [89]. Among 29 transfusion-dependent patients at baseline (requiring ≥ 2 units of RBC transfusions in the 30 days preceding the first dose of momelotinib), 15 (51.7%) were transfusion-independent for ≥ 8 weeks, and Hb increased by 2 g/dL or more for ≥ 8 weeks in 3 out of 11 non-transfusion-dependent patients (27%) [89]. When baseline transfusion-dependence was defined as the need for ≥ 6 units of RBC transfusions in the 12 weeks preceding the first dose of momelotinib (at least one transfusion during the 28 days before the first dose), 4/19 (21%) patients achieved transfusion-independence for ≥ 12 weeks [89]; the cohort that achieved 12-week transfusion-independence was smaller, but the responses were more durable compared to the larger subgroup that remained transfusion-independent for ≥ 8 weeks [89].

The results of a translational phase 2 study of 41 MF patients who were transfusion-dependent and received treatment with momelotinib were recently reported [67]. Forty one percent of these patients (17/41) achieved transfusion-independence (defined as absence of RBC transfusion for 12 weeks or more at any time on the study) [67]. The transfusion-independent cohort included 14 patients (34%) who met the criteria by week 24. Furthermore, the requirement for transfusions decreased by ≥ 50% for ≥ 8 weeks in 21/27 (78%) of the transfusion-dependent patients [67]. The transfusion-independence response rate at any time during the study was nearly 50% in the evaluable population after more than 12 weeks of follow-up [67].

SIMPLIFY-1 [95] and SIMPLIFY-2 [96] were two randomized phase 3 trials conducted in patients with primary or secondary MF; in both trials, patients were allowed to cross over to the momelotinib arm after the 24-week response assessment time point. In the SIMPLIFY-1 trial, momelotinib was evaluated head-to-head against ruxolitinib in 432 JAK-inhibitor naïve patients (randomized 1:1) with high-risk, intermediate-2 or symptomatic intermediate-1 risk MF and platelet counts ≥ 50 × 10^9^/L for 24 weeks (double-blind dosing period) [95]. Regarding the secondary endpoint of anemia benefit in the SIMPLIFY-1 trial, at 24 weeks, 66.5% of the MF patients on momelotinib achieved or maintained transfusion independence (defined as absence of RBC transfusion and no Hb level below 8 g/dL in the previous 12 weeks) versus 49.3% on the ruxolitinib arm (nominal *P* < 0.001); at 48 weeks, the corresponding transfusion-independence rates for patients on momelotinib from the outset and those who crossed over from the ruxolitinib arm to momelotinib after the 24-week randomized treatment period were 75% and 67%, respectively [91]. In SIMPLIFY-1, at baseline, the respective percentages of transfusion-independent patients were 68.4% and 70.0% for momelotinib and ruxolitinib, respectively [95]. Further retrospective analysis of the SIMPLIFY-1 data showed that at week 24, the rate of transfusion-independence was substantially higher in the momelotinib arm compared to the ruxolitinib arm, regardless of Hb levels (and baseline platelet count) [97]. For example, for baseline Hb < 8 g/dL, 29% versus 18% of the patients achieved transfusion independence with momelotinib versus ruxolitinib, respectively, at week 24 [97]. For baseline Hb < 10 g/dL and Hb < 12 g/dL, the respective transfusion-independence rates were 47% vs. 27% and 62% vs. 37%, for momelotinib and ruxolitinib, respectively, at week 24 [97]. In SIMPLIFY-1, the rate of transfusion-dependence was 30.2% vs. 40.1% for the momelotinib and ruxolitinib arm, respectively, at week 24 (nominal *P* = 0.019); at baseline, 24.7% and 24.0% of the patients were transfusion-dependent, respectively [95]. The median RBC transfusion rate was 0 units/month for momelotinib and 0.4 units/month for ruxolitinib through week 24 (nominal *P* < 0.001) [95]. The average cumulative number of transfused RBC units at any time point was nearly one-half in patients treated with momelotinib (HR = 0.522, *P* < 0.0001) as compared to ruxolitinib in models with and without patients' baseline characteristics as covariates [98]. Kaplan–Meier function estimates of the data showed that the median duration of transfusion independence was not reached in the SIMPLIFY-1 trial after follow-up of more than 3 years for both patients who began with momelotinib and those who crossed over from ruxolitinib to momelotinib [91]. Further analysis of the anemia benefit endpoints in SIMPLIFY-1 demonstrated that 83% of the patients receiving momelotinib required ≤ 4 units of RBCs versus 62% on ruxolitinib (*P* < 0.0001) during the 24 weeks of randomized treatment [98]. Importantly, retrospective analysis of the transfusion data (on the basis of a zero-inflated negative binomial covariate model), acquired in the SIMPLIFY-1 trial, showed that the odds of momelotinib-treated patients remaining transfusion-independent were 9.3 times higher (*P* < 0.0001) as compared to ruxolitinib-treated patients [98].

In the multinational SIMPLIFY-2 trial, the efficacy of momelotinib was compared to BAT (89% of the patients received ruxolitinib as BAT) in 156 anemic or thrombocytopenic MF patients, randomized 2:1, over a 24-week open-label treatment phase [96]. In the SIMPLIFY-2 trial, the patients had to have been previously exposed to ruxolitinib for 28 days or more and required either RBC transfusions while on ruxolitinib or a dose reduction to < 20 mg twice daily due to grade ≥ 3 anemia, thrombocytopenia or bleeding [96]. At week 24, 43% of the patients treated with momelotinib were transfusion-independent versus 21% in the BAT/ruxolitinib arm (nominal *P* = 0.0012) [96]. At 48 weeks, the corresponding transfusion-independence rates were 55% and 40% for patients on momelotinib from the outset and those who crossed over from the BAT arm to momelotinib after the 24-week randomized period, respectively [91]. Over the entire treatment period, 40% of the momelotinib-treated patients did not require RBC transfusions as compared to 27% in the BAT arm [96]. In addition, the rate of transfusion-dependence was 50% versus 64% for momelotinib and ruxolitinib, respectively, at week 24 (nominal *P* = 0.10) [96]. Kaplan–Meier function estimates of the SIMPLIFY-2 data showed that the median duration of transfusion independence at any time during the study was more than one year with momelotinib [91]. In SIMPLIFY-2, the median rate of RBC transfusions was 0.5 units/month for momelotinib and 1.2 units/month for BAT/ruxolitinib through week 24 (nominal *P* = 0.39) [96].

Other analyses of the combined dose-intensity data from the SIMPLIFY-1 and SIMPLIFY-2 trials showed that near-maximal momelotinib dose intensity (200 mg once daily) was administered throughout the 24-week treatment period in 90% of the momelotinib-randomized patients who ranged from JAK-inhibitor naïve to individuals with intermediate- /high-risk MF previously treated with a JAK-inhibitor; and 85% of the patients continued on the same dose during the extended treatment phase thereafter [99]. In contrast to the durable dose intensity of momelotinib, which is attributed to its low myelo-suppressive potential and demonstrable anemia benefit, low and diminishing dosing was required for ruxolitinib due to its hematologic toxicity (only 32% of the patients were treated with the maximum recommended ruxolitinib dose during week 24 of the randomized treatment period) [99]. However, it is well known that the efficacy of ruxolitinib regarding spleen responses is dose-dependent [100]. Importantly, about 85% of the patients originally randomized to ruxolitinib who crossed over to momelotinib after 24 weeks of participation in the randomized phases of the SIMPLIFY-1 and SIMPLIFY-2 trials (including patients who received considerably reduced doses of ruxolitinib) were able to receive the maximum dose of momelotinib for an extended period of time. The aforementioned data further underscore the differentiated biological profiles of the two JAK inhibitors and confirm the unique benefits that momelotinib may provide in patients who previously experienced hematological toxicity from ruxolitinib [99]. Furthermore, during the extended treatment period of the SIMPLIFY-1 trial, a significantly lower rate of anemia (grade 3/4) was reported in patients treated with momelotinib (4.7%) vs. ruxolitinib (18.5%) in the preceding randomized period [101]. During the extended treatment period of SIMPLIFY-2, the rate of anemia (grade 3/4) was 3.1%  for patients treated with momelotinib from the onset, and 5.0% in the cohort that crossed over from  BAT to momelotinib [101]. These findings underscore the favorable hematologic toxicity profile and limited myelosuppression with momelotinib. Furthermore, retrospective analyses of the data collected from the SIMPLIFY-1 and SIMPLIFY-2 trials demonstrated that the anemia benefit of momelotinib is maintained in both frontline and second-line thrombocytopenic patients [102].

Besides the benefits of momelotinib with respect to anemia/transfusion-dependence and its ability to also effectively address the other two hallmarks of MF (splenomegaly and constitutional symptoms) [103], momelotinib conferred notable survival outcomes in both JAK inhibitor-naïve and ruxolitinib-pretreated patients. In particular, in the SIMPLIFY-1 trial, patients who were treated with ruxolitinib during the initial randomization period and crossed over to momelotinib thereafter had a median OS of 53.1 months, whereas the median OS had not been reached for the patients originally randomized to momelotinib (HR = 0.99, *P* = 0.97) [91]. In the SIMPLIFY-2 trial, the patients who were randomized to BAT (primarily ruxolitinib) for 24 weeks and then crossed over to momelotinib had a median OS of 37.5 months; the patients who were originally randomized to momelotinib had a median OS of 34.3 months (HR = 0.96, *P* = 0.86) [91]. In the SIMPLIFY trials, OS of the patients was followed for a maximum of approximately 5 years and a median of 2.9 years and 2.3 years for SIMPLIFY-1 and SIMPLIFY-2, respectively. Further retrospective analyses of the two SIMPLIFY studies revealed additional OS advantages for patients who achieved transfusion-independence [104]. In SIMPLIFY-1, the patients who achieved transfusion-independence with momelotinib treatment at week 24 did not reach a  median OS, and the 3-year survival was 80% (HR = 0.30, *P* = 0.0001; relative to momelotinib-treated patients who did not become transfusion-independent) [104]. A similar trend towards a more favorable OS was observed in the momelotinib-treated patients who became transfusion-independent (at week 24) in SIMPLIFY-2 (HR = 0.57, *P* = 0.0652) as compared to non-responders regarding transfusion-independence [104]. These findings further underscore a potentially prognostically important association between transfusion-independence at week 24 with survival advantage in MF patients receiving momelotinib.

Currently, momelotinib is being evaluated in comparison to danazol (2:1) in the double-blind, randomized phase 3 registration-track MOMENTUM trial (NCT04173494) in 180 patients (trial accrual was completed), with primary or secondary MF, who were anemic (Hb < 10 g/dL) and symptomatic (total symptom score ≥10); and had been previously treated with an approved JAK inhibitor. After the 24-week randomized period of the trial, patients in the danazol arm could cross over to momelotinib [90]. In this trial, while the primary endpoint is the proportion of patients achieving a reduction in the total symptom score ≥ 50% from baseline at week 24, key secondary endpoints include the proportion of transfusion-independent patients (defined as not requiring a RBC transfusion for ≥ 12 weeks and having Hb ≥ 8 g/dL at week 24) and the proportion of patients who achieve spleen volume reduction ≥ 35% from baseline at week 24; and other measures of anemia benefit, such as Hb improvement and cumulative transfusion burden [90]. The results of the MOMENTUM trial are eagerly awaited and may lead to regulatory approval of momelotinib. Danazol was selected as an appropriate comparator to momelotinib in the MOMENTUM trial because it is one of the recommended agents to treat anemia in MF patients according to the guidelines of the National Comprehensive Cancer Network [105] and the European Society of Medical Oncology [106].

## Conclusions

Momelotinib is a highly promising, orally bioavailable, investigational agent that targets and selectively inhibits JAK1/JAK2 and ACVR1, a serine/threonine kinase strongly implicated in iron homeostasis. Momelotinib’s mechanism of action uniquely positions it amongst approved and late-stage JAK inhibitors to be able to significantly alleviate the inflammation-driven, iron-restricted anemia of MF and eliminate/prevent RBC transfusion dependence in a significant proportion of MF patients besides treating the other two cardinal features of MF (splenomegaly and constitutional symptoms).

Furthermore, momelotinib is minimally myelosuppressive and, along with pacritinib, could help patients with “cytopenic/myelodepletive” MF, especially where anemia is prominent. According to a recent systematic review and network meta-analysis that were performed on the data from seven first-line and second-line randomized phase 3 trials (COMFORT-1/2, JAKARTA, PERSIST-1/2, and SIMPLIFY-1/2) that assessed the four JAK inhibitors (ruxolitinib, fedratinib, pacritinib, and momelotinib), no statistically significant differences were found between ruxolitinib, fedratinib and pacritinib regarding grade 3/4 anemia events on the basis of odds ratios (ORs) for toxicity endpoints; however, momelotinib had a significantly lower OR compared to the other JAK inhibitors regarding anemia events (OR was 0.32 for momelotinib versus 1, 0.85, and 0.82 for ruxolitinib, fedratinib and pacritinib, respectively [107]. Furthermore, the same study demonstrated that momelotinib was not statistically significantly different regarding spleen volume reduction compared to ruxolitinib and fedratinib (on the basis of data from first-line clinical trials) [107].

The current development strategy for momelotinib focuses on the second-line setting, a space with a critical unmet need given that nearly all MF patients will become anemic over the course of the disease; however, it is possible to envision first-line use of momelotinib as well, particularly in significantly anemic patients, owing to the non-inferiority of spleen response compared to ruxolitinib in the SIMPLIFY-1 study. In addition, as shown in the retrospective analyses of the data collected in the SIMPLIFY-1 and SIMPLIFY-2 trials, momelotinib was efficacious in both frontline and second-line thrombocytopenic patients, and the majority of the patients who crossed over to momelotinib after randomization in SIMPLIFY-1 and SIMPLIFY-2 were able to receive the maximum momelotinib dose for an extended period of time. These results testify to the agent’s good tolerability with occasional side effects of low-grade nausea and peripheral neuropathy, thereby allowing sustained dosing of momelotinib and prolonged clinical benefits. Indeed, the median duration of transfusion-independence was not reached after more than 3 years in patients who achieved transfusion-independence in SIMPLIFY-1, and the median duration of transfusion-independence on momelotinib was more than one year in SIMPLIFY-2. As detailed above, momelotinib may also have overall survival benefits in frontline and second-line MF patients; however, no statistically significant difference was found between the two arms of SIMPLIFY-1 (momelotinib versus ruxolitinib) with respect to leukemic transformation [103].

Combination therapies in advanced clinical development for MF [108], such as those of ruxolitinib with luspatercept (activin receptor IIB ligand trap/erythroid maturation agent) or pelabresib (bromodomain and extra-terminal protein inhibitor) may represent therapeutic alternatives to momelotinib, assuming all are eventually approved. For example, ruxolitinib in combination with pelabresib could be envisioned as a viable treatment for MF patients with good blood counts whereas MF patients with anemia could be treated with ruxolitinib in combination with luspatercept or momelotinib monotherapy. However, momelotinib retains the appeal of being a single agent with great potential to address all three major manifestations of the disease. Momelotinib may receive regulatory approval in the near future as a treatment for MF patients with anemia.

## Data Availability

Not applicable.

## Abbreviations

ACVR1: Activin A receptor, type 1
ALK2: Activin receptor-like kinase-2
Allo-HSCT: Allogeneic hematopoietic stem cell transplantation
AML: Acute myeloid leukemia
BAT: Best available therapy
BMP: Bone morphogenetic protein
BMP2: Bone morphogenetic protein 2
BMP6: Bone morphogenetic protein 6
BMP RE: Bone morphogenetic protein response element
DIPSS: Dynamic International Prognostic Scoring System
EPO: Erythropoietin
ERFE: Erythroferrone
H: Hepcidin
*HAMP*: Hepcidin gene
Hb: Hemoglobin
HFE: Homeostatic iron regulator
HJV: Hemojuvelin
HR: Hazard ratio
IC_50_: Half maximal inhibitory concentration
IL-6: Interleukin-6
IPSS: International Prognostic Scoring System
IRAK1: interleukin-1 receptor-associated kinase 1
JAK: Janus kinase
LSECs: Liver sinusoidal endothelial cells
MF: Myelofibrosis
MIPSS70: Mutation-Enhanced International Prognostic Scoring System 70
MMB: Momelotinib
MYSEC-PM: Myelofibrosis SECondary to PV and ET-Prognostic Model
OR: Odds ratio
OS: Overall survival
PMF: Primary myelofibrosis
pSTAT3: Phosphorylated STAT3
RBC: Red blood cell
SMAD: Small-mothers-against-decapentaplegic
STAT: Signal transducer and activator of transcription
STAT3 RE: STAT3 response element
Tf: Transferrin
TfR1: Transferrin receptor-1
TfR2: Transferrin receptor-2
TGF-β: Transforming growth factor beta
TMPRSS6: Transmembrane protease, serine 6
TSS: Total Symptom Score
